# A subset of human plasmacytoid dendritic cells expresses CD8α upon exposure to herpes simplex virus type 1

**DOI:** 10.3389/fmicb.2015.00557

**Published:** 2015-06-02

**Authors:** Philipp Schuster, Sabrina Thomann, Maren Werner, Jörg Vollmer, Barbara Schmidt

**Affiliations:** ^1^Institute of Medical Microbiology and Hygiene, University of Regensburg, Regensburg, Germany; ^2^Institute of Clinical and Molecular Virology, Friedrich-Alexander-Universität Erlangen-Nürnberg, Erlangen, Germany; ^3^Nexigen, Cologne, Germany

**Keywords:** dendritic cells, plasmacytoid, virus, HSV, human, murine

## Abstract

Classical and plasmacytoid dendritic cells (DC) play important roles in the defense against murine and human infections with herpes simplex virus (HSV). So far, CD8α expression has only been reported for murine DC. CD8α^+^ DC have prominent cross-presenting activities, which are enhanced by murine CD8α^+^ PDC. The human orthologue of murine CD8α^+^ DC, the CD141 (BDCA3)^+^ DC, mainly cross-present after TLR3 ligation. We report here the serendipitous finding that a subset of human PDC upregulates CD8α upon HSV-1 stimulation, as shown by gene array and flow cytometry analyses. CD8α, not CD8ß, was expressed upon exposure. Markers of activation, migration, and costimulation were upregulated on CD8α-expressing human PDC. In these cells, increased cytokine and chemokine levels were detected that enhance development and function of T, B, and NK cells, and recruit immature DC, monocytes, and Th1 cells, respectively. Altogether, human CD8α^+^ PDC exhibit a highly activated phenotype and appear to recruit other immune cells to the site of inflammation. Further studies will show whether CD8α-expressing PDC contribute to antigen cross-presentation, which may be important for immune defenses against HSV infections *in vitro* and *in vivo*.

## Introduction

Since Ralph Steinman first described a new subset of cells characterized by tree-like processes in 1973 (Steinman and Cohn, 1973), knowledge about dendritic cells (DC) in mice and humans has grown exponentially. These cells were originally identified as important players in the defense against “foreign” pathogens, but it turns out that they are similarly crucial in initiating immune responses against tumor-associated antigens (Vacchelli et al., 2013). Immature DC engulf extracellular antigens, but in the absence of appropriate danger signals, they induce peripheral tolerance. Only after appropriate activation, DC release cytokines and chemokines, undergo a maturation process, and migrate toward secondary lymphatic tissues to induce cytotoxic responses by other immune cells (Palucka and Banchereau, 2012; Merad et al., 2013).

In this perspective paper, we will focus on the role of CD8α-expressing DC. CD8 serves as useful subset marker for murine DC, which are highly efficient in cross-presenting foreign, self, and—most likely—tumor-associated antigens, although evidence is lacking that CD8 expression plays any role in the development and function of these cells (Shortman and Heath, 2010). So far, CD8 expression on human DC has not been reported (Naik, 2008). However, we report here a serendipitous finding of CD8α expression on human plasmacytoid dendritic cells (PDC) after stimulation with herpes simplex virus type 1 (HSV-1), which characterizes a highly activated subset of PDC. We will discuss how the knowledge about CD8α-expressing murine DC may translate into functions of CD8α-expressing human PDC. For the background of this topic, the reader is referred to excellent review articles by respected colleagues (Villadangos and Young, 2008; Shortman and Heath, 2010; Joffre et al., 2012; Nierkens et al., 2013; Bedoui and Greyer, 2014; Boltjes and van Wijk, 2014).

## Murine CD8α^+^ DC: Prominent Cross-presentation

In the murine system, several different DC populations exist: lymphoid-organ resident CD8α^+^ or CD8α^–^ DC; migratory dermal CD103^+^ or CD103^–^ DC, which migrate to lymphatic tissue; Langerhans cells in the skin; inflammatory DC, which develop from monocytes; and PDC (Vremec et al., 1992; Bursch et al., 2007; Ginhoux et al., 2007, 2009; Leon et al., 2007; Poulin et al., 2010; Joffre et al., 2012). CD8α is a marker for lymphoid tissue-resident DC, which make up roughly 20% of spleen DC and 70% of thymic DC, whereas only 0.2% of peripheral blood mononuclear cells are CD8α^+^ DC (Crowley et al., 1989; Vremec et al., 2000; Donnenberg et al., 2001; Henri et al., 2001; Shortman and Heath, 2010). These cells express a CD8αα homodimer rather than the CD8αß heterodimer on T cells (Vremec et al., 1992, 2000). Precursors of CD8α^+^ DC may lack CD8 expression (Martinez del Hoyo et al., 2002). Apart from the classical CD8α^+^ DC population, this molecule is expressed by murine PDC in the spleen (O’Keeffe et al., 2002) and other migratory DC after activation (Anjuere et al., 1999, 2000; Merad et al., 2000; Henri et al., 2001). Mice with a knock-out for interferon regulatory factor (IRF) 8 neither develop CD8α^+^ DC nor PDC (Schiavoni et al., 2002; Aliberti et al., 2003; Tsujimura et al., 2003), whereas Batf3-deficient mice are only deficient in CD8α^+^ DC (Hildner et al., 2008; Edelson et al., 2010).

Amongst other receptors, the murine CD8α^+^ DC subset expresses CD11c, CD24, CD36, Necl2, MHC-II, the integrin CD103, the lectins CD205, CLEC9A, CLEC12A, and langerin (CD207) (Shortman and Heath, 2010). CLEC9A and CD36 are both involved in recognizing late apoptotic or necrotic cells (Albert et al., 1998; Caminschi et al., 2008; Huysamen et al., 2008; Sancho et al., 2009). Murine CD8α^+^ DC also express TLR3 and TLR9 (Edwards et al., 2003), and respond to TLR stimulation with proinflammatory IL-12 secretion and at least some type I interferon production (Hochrein et al., 2001). Upon stimulation, CD8α^+^ DC upregulate costimulatory markers CD40, CD80, and CD86 as well as CD25, CD62L, and MHC-II (Wilson et al., 2003).

CD8α^+^ DC are most efficient in antigen cross-presentation, a process in which extracellular antigen is not presented on MHC-II to CD4^+^ T cells, but instead shunted to MHC-I with subsequent induction of CD8^+^ T cells. Cross-presentation occurs through the cytosolic or vacuolar pathway (Joffre et al., 2012). The former involves proteasomal degradation with subsequent transport of peptides into the endoplasmic reticulum via transporter associated with antigen processing 1 (TAP), whereas the latter is based on lysosomal proteolysis with subsequent loading of peptides onto MHC-I molecules (Joffre et al., 2012).

In this respect, CD8α^+^ DC, but not CD8α^–^ DC, were shown to cross-prime using a TAP-dependent pathway (den Haan et al., 2000; Pooley et al., 2001; Schnorrer et al., 2006; Lin et al., 2008). CD8α^+^ DC have been reported to selectively engulf dying cells *in vitro* and *in vivo* and present on MHC-I via a proteasome-dependent pathway (Iyoda et al., 2002; Schulz and Reis e Sousa, 2002). In these cells, endosomal acidification is limited (Savina et al., 2009), which fosters limited antigen degradation and efficient transport of the antigen to the cytosol (Delamarre et al., 2005). Overexpression of MHC-I loading complexes (Dudziak et al., 2007) by CD8α^+^ DC and expression of chemokine receptor XCR1, whose ligand XCL1 is secreted by activated CD8^+^ T cells, contribute to antigen cross-presentation and differentiation of cytotoxic T cells (Dorner et al., 2009).

In HSV infections, CD8α^+^ DC are able to present viral antigens and prime naïve CD4^+^ and CD8^+^ T cells, which appears to be mediated by cross-presentation (Allan et al., 2003; Smith et al., 2003; Belz et al., 2004a,b; Wilson et al., 2006; Bedoui et al., 2009; Lee et al., 2009). It is still a matter of debate how the viral antigen is transported from peripheral infected tissue to the lymphoid-resident CD8α^+^ DC. In this process, mainly other (migratory) DC are reported to be involved (Zhao et al., 2003; Carbone et al., 2004; Allan et al., 2006; Bedoui et al., 2009; Jirmo et al., 2009). These migratory DC either capture viral antigens or are infected within the peripheral tissue, although reduced migratory capacity has been reported for HSV-infected DC (Jones et al., 2003; Eidsmo et al., 2009; Puttur et al., 2010). The transfer of viral antigen can occur via exosomes, gap junctions, or uptake of apoptotic material following death of migratory DC (Thery et al., 2009; Mazzini et al., 2014). Another option is “crossdressing”, i.e., the transfer of preformed MHC-I complexes loaded with peptides from infected cells to murine DC via secreted membrane vesicles or transfer of membrane fragments (trogocytosis) (Thery et al., 2009; Wakim and Bevan, 2011; Joffre et al., 2012).

## Murine CD8α^+^ PDC: Cross-presentation Help

Murine PDC were identified in the spleen of mice (Asselin-Paturel et al., 2001; O’Keeffe et al., 2003). Amongst other surface receptors, they express Ly6C, B220, and CD11c. Upon stimulation, type I interferons—and to a minor extent IL-12—are induced, and costimulatory markers CD40, CD69, CD80, and CD86 are upregulated (Asselin-Paturel et al., 2001; O’Keeffe et al., 2002; Lund et al., 2003). Unstimulated murine PDC express CD8α only to a minor extent, while exposure to CpG or viruses enhances expression of this molecule (Nakano et al., 2001; O’Keeffe et al., 2002, 2003). When CD8α^+^ and CD8α^–^ PDC were separated and subsequently stimulated, they did not differ in cytokine production (O’Keeffe et al., 2002).

A few publications report TAP-dependent cross-presentation of soluble and particulate antigen by murine PDC after TLR ligation (Shinohara et al., 2006; Mouries et al., 2008; Kool et al., 2011). The majority of authors, however, deny cross-presentation by murine PDC (Chung et al., 2005; Janssen et al., 2006; Sapoznikov et al., 2007; GeurtsvanKessel et al., 2008; Reboulet et al., 2010; Hennies et al., 2011). *In vitro* stimulation of murine PDC with HSV-1 or influenza allowed priming of CD8^+^ T cells (Belz et al., 2004a). In *in vivo* HSV-1 infections, however, PDC do not participate in active cross-presentation (Allan et al., 2003; Lee et al., 2009; Swiecki et al., 2013). Still, murine PDC appear to be important in enhancing cross-presentation by other DC. An explanation of this phenomenon could be that type I interferons increase cross-presentation by decreasing antigen degradation in endocytic compartments and stimulating the survival of CD8α^+^ DC (Diamond et al., 2011; Fuertes et al., 2011; Lorenzi et al., 2011). In this respect, depletion of murine PDC was reported to impair CTL-mediated HSV-1 eradication in a CD2-, CD40L-, and type I interferon-dependent manner (Yoneyama et al., 2005). Also in the lymphocytic choriomeningitis model, virus-induced type I interferons were required for cross-priming of CD8^+^ T cells (Le Bon et al., 2003). When PDC were depleted in CLEC4C-DTR mice, PDC proved to be important for inducing CD8^+^ T cell responses in systemic HSV-1 and HSV-2 infections (Swiecki et al., 2013). Further functions of PDC in murine HSV-1 and HSV-2 infections are reviewed in (Schuster et al., 2011).

## Human Orthologue of CD8α^+^ DC: Cross-presentation Following Activation

The conventional human blood DC population consist of three subsets specifically expressing CD1c (BDCA1), CD16, or CD141 (BDCA3) (Dzionek et al., 2000; MacDonald et al., 2002). Evidence is accumulating that the CD11c^+^ CD141^+^ DC subset represents the human orthologue of murine CD8α^+^ DC. These cells can be detected in lymphatic tissues such as lymph nodes, tonsils, bone marrow, spleen, and also liver (Galibert et al., 2005; Lindstedt et al., 2005; Velasquez-Lopera et al., 2008; Bamboat et al., 2009; Poulin et al., 2010). Genome-wide expression analyses revealed a similar transcriptomal signature between CD141^+^ human DC and murine CD8α^+^ DC (Robbins et al., 2008). Both subsets express Necl2 (Galibert et al., 2005), CLEC9A (Caminschi et al., 2008; Huysamen et al., 2008; Sancho et al., 2009; Jongbloed et al., 2010; Schreibelt et al., 2012), TLR3 (Edwards et al., 2003; Lindstedt et al., 2005; Jongbloed et al., 2010), as well as CD207, Batf3, and IRF8 (Poulin et al., 2010). BDCA3^+^ DC also express the chemokine receptor XCR1 and respond to respective ligands (Bachem et al., 2010; Crozat et al., 2010). Similar to murine CD8α^+^ DC, human BDCA3^+^ DC respond to TLR3 ligation with production of lambda interferons (Lauterbach et al., 2010). In contrast to murine CD8α^+^ DC, human BDCA3^+^ DC do not express TLR9 (Jongbloed et al., 2010).

Lymphoid tissue-derived human BDCA3^+^ DC were shown to be at least equivalent to other human DC subsets in cross-presenting soluble or cell-associated antigens, even in the absence of activation (Segura et al., 2012, 2013). This process can be enhanced by stimulation with TLR3 ligands, inducing superior cross-presenting activity by blood-derived BDCA3^+^ DC with induction of CD8^+^ T cell responses (Poulin et al., 2007; Bachem et al., 2010; Crozat et al., 2010; Jongbloed et al., 2010). There is evidence that cross-presentation by myeloid DC plays a role in human herpes virus infections (Bosnjak et al., 2005), but the importance of BDCA3^+^ DC needs to be further clarified.

## Human CD8α^+^ PDC: Cross-presentation Help Following Viral Activation?

In 1999, two independent groups identified human PDC as major producers of type I interferons in the blood (Cella et al., 1999; Siegal et al., 1999). Amongst other receptors, PDC express BDCA2 and BDCA4, MHC-II, the lymph node-homing receptors CD62L and CCR7 (CD197), and costimulatory molecules (CD40, CD80, CD86, CD270, CD274, CD275) (Cella et al., 2000; Dzionek et al., 2000; Ito et al., 2007; Jaehn et al., 2008; Donaghy et al., 2009; Schuster et al., 2010, 2011; Cabezon et al., 2011). PDC recognize single-stranded RNA and CpG molecules via TLR7 and TLR9, respectively (Kadowaki et al., 2001).

Whether human PDC can cross-present soluble or particulate antigens is still a matter of debate. Viral antigen derived from influenza, recombinant vaccinia, tick-borne encephalitis or human immunodeficiency type I virus infection was taken up into recycling endosomes, loaded onto MHC-I molecules, and presented to CD8^+^ T cells (Fonteneau et al., 2003a,b; Hoeffel et al., 2007; Di Pucchio et al., 2008; Lui et al., 2009; Mittag et al., 2011; Tel et al., 2012). In addition, antigen loaded on synthetic microparticles or soluble tumor-associated antigen was presented to CD8^+^ T cells by exposed PDC (Tel et al., 2010; Guillerme et al., 2013; Segura et al., 2013). In contrast, other groups report no or only minor cross-presenting capacities of human PDC (Schnurr et al., 2005; Bachem et al., 2010; Crozat et al., 2010).

An early report by Fitzgerald-Bocarsly described the “interferon-producing cells” as being important for the lysis of HSV-infected fibroblasts (Feldman et al., 1992). PDC infiltrate herpetic lesions in the genital tract and tightly colocalize with NK and T cells (Donaghy et al., 2009). HSV-stimulated human PDC induce migration of activated T and NK cells via chemokine secretion (Megjugorac et al., 2004), and contribute to the activation of NK cells via IFNα- and TNFα-dependent mechanism (Vogel et al., 2014). In addition, HSV-exposed PDC were shown to prime IL-10 and IFN-γ production by cytotoxic regulatory CD4^+^ T cells (Kadowaki et al., 2000; Kawamura et al., 2006).

So far, expression of CD8 on human PDC has not been reported. Since the expression of this molecule on the surface of human PDC may define new and yet unknown capacities of these cells, we investigated whether PDC might upregulate these molecules upon viral stimulation. Recently, we analyzed the expression profile of human PDC, which were purified from PBMC of six donors. After exposure to IL-3 or IL-3 plus UV-inactivated HSV-1 (HSV_UV_), RNA was extracted from these cells and hybridized to a Human Genome U133 Plus 2_0 Array (Affymetrix, Santa Clara, CA, USA) (Schuster et al., 2010). In these analyses, we focused on the expression and regulation of surface receptors on PDC. Notably, the signal for CD8α expression increased from 57.2 to 100.1, which was slightly above the arbitrary threshold of 95, reflecting the expression signal of TLR9. In contrast, three probe sets for CD8β remained below this threshold. These data suggested a potential expression of CD8α on PDC upon stimulation with HSV_UV_.

**FIGURE 1:**
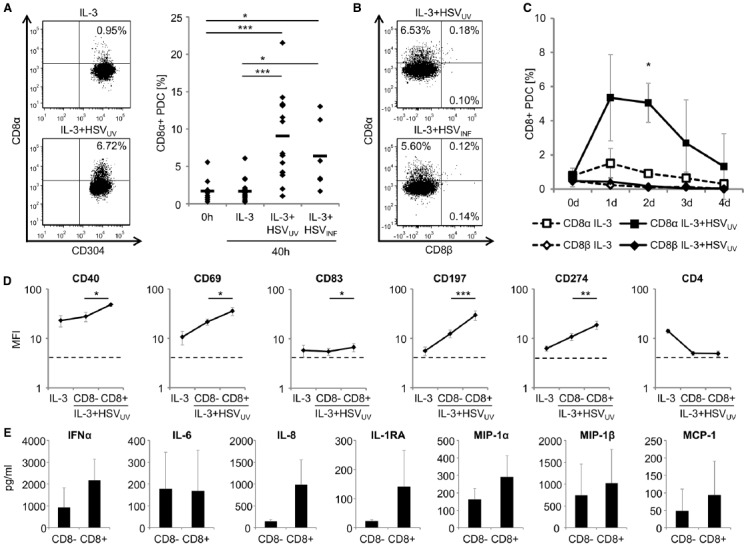
**Characterization of CD8α-expressing human plasmacytoid dendritic cells (PDC). (A)** Expression of CD8α on human PDC, as evaluated by flow cytometry within PBMC immediately after cell isolation (0 h) (*n* = 9) and after cultivation of purified PDC in the presence of IL-3 (10 ng/ml), IL-3 plus UV-inactivated herpes simplex virus type 1 (HSV_UV_) (*n* = 16) or infectious HSV-1 (HSV_INF_) (*n* = 6) (1 × 10^6^ plaque-forming units/ml) for 40 h. One representative example of PDC exposed to IL-3 (upper left panel) and IL-3 plus HSV_UV_ (lower left panel) and data of all donors including mean values (bars) are shown (right panel). **(B)** Representative expression of CD8α, not CD8β, on human PDC after exposure to IL-3 plus HSV_UV_ (upper panel, *n* = 4) or HSV_INF_ (lower panel, *n* = 3) for 40 h. **(C)** Kinetics of CD8α (*n* = 4) and CD8β (*n* = 3) expression after exposure of human PDC to IL-3 or IL-3 plus HSV_UV_ for 4 days. Data are presented as mean and standard deviation. **(D)** Expression of markers for costimulation (CD40, CD274), activation (CD69), maturation (CD83), and migration (CD197) on CD8α^+^ and CD8α^–^ human PDC after exposure to IL-3 plus HSV_UV_ for 40 h. The dotted lines represent isotype controls. For statistical analysis, MFI values were transformed logarithmically to obtain normal distribution. Expression of CD4 as well as CD2, CD46, CD80, and CD123 (data not shown) was not significantly different between the two PDC subsets. Mean and standard deviation of four different donors (except for CD274, *n* = 3). **(E)** After stimulation of human PDC with IL-3 plus HSV_UV_ for 40 h, cells were harvested and then separated using a CD8 cell isolation kit (Miltenyi Biotec, Bergisch-Gladbach, Germany). After stimulation with IL-3 or IL-3 plus HSV_UV_ for another 20 h, different cytokines were analyzed in the cell culture supernatants using a multiplex cytokine and chemokine panel on a luminex platform (Invitrogen/Life Technologies, Darmstadt, Germany, and Affymetrix/ebioscience, Frankfurt, Germany). Mean and standard deviation of three different donors. *p < 0.05, **p < 0.01, ***p < 0.001.

To corroborate these data, we isolated PDC from a total of 15 different donors, and investigated CD8α expression on these cells in independent experiments after exposure to IL-3 (*n* = 16), IL-3 plus HSV_UV_ (*n* = 16), or IL-3 plus infectious HSV-1 (HSV_INF_, *n* = 6) for 40 h. Flow cytometry confirmed a distinct expression of CD8α on a subset of HSV_UV_- and HSV_INF_-exposed PDC (Figure 1A). After stimulation with HSV_UV_ or HSV_INF_, the percentage of CD8α-expressing PDC was significantly higher compared to PDC within freshly isolated PBMC (*n* = 9) (*p* < 0.001 for HSV_UV_ and *p* < 0.05 for HSV_INF_, unpaired *t*-test) and purified PDC that were cultivated in the presence of IL-3 only (*p* < 0.001 for HSV_UV_ and *p* < 0.05 for HSV_INF_, paired *t*-test) (Figure 1A). CD8α expression was not different between HSV_UV_- and HSV_INF_-exposed PDC (*p* = 0.27, n.s.). When we stained in parallel for CD8α and CD8β expression, we confirmed expression of CD8α by flow cytometry, while CD8β was neither detected on PDC exposed to HSV_UV_ (*n* = 4) nor HSV_INF_ (*n* = 3) (Figure 1B). These data indicated that PDC did not express a heterodimeric CD8αβ receptor upon stimulation. In further analyses, we investigated the kinetics of CD8α expression (*n* = 4). After exposure to HSV_UV_, the percentage of CD8α-expressing cells increased by day 1, but reached significance by day 2 post stimulation, compared to PDC cultivated with IL-3 alone (*n* < 0.05, paired *t*-test). Expression of CD8β was not detected at any of the time points analyzed (*n* = 3) (Figure 1C).

To find out in how far CD8α^+^ and CD8α^–^ PDC differed from each other, we analyzed the expression of cell surface markers for costimulation (CD40, CD274), activation (CD69), maturation (CD83), and migration (CD197) on these two subsets. All these markers were significantly upregulated on CD8α^+^ PDC compared to CD8α^–^ PDC (*p* < 0.05, paired *t*-test) (Figure 1D), while five other surface molecules (CD2, CD4, CD46, CD80, and CD123) were not differently regulated after HSV_UV_ stimulation. These data suggested that the subset of CD8α^+^ PDC was particularly activated. Eventually, we exposed PDC of three donors to HSV_UV_ for 40 h, separated these cells using a CD8 cell isolation kit, and exposed the CD8α^+^ and CD8α^–^ PDC to HSV_UV_ for another 20 h. Subsequently, cell culture supernatants were analyzed using a multiplex cytokine bead assay. Of a total of 25 cytokines, we found IFN-α, IL-8, IL-1RA, MIP-1α, MIP-1β, and MCP-1 upregulated in CD8α-expressing PDC. In contrast, IL-6 secretion was not different between the two subsets, and other cytokines were either not induced (IL-1β, IL-17, IFN-γ, GM-CSF, MIG, RANTES) or expressed only at very low levels (IL-2, IL-4, IL-5, IL-7, IL-12p40, IL-13, IL-15, eotaxin) (Figure 1E). IFN-α and IL-6 enhance T cell, B cell, and NK cell development and function; IL-8 recruits T cells and induces their degranulation; IL-1RA inhibits IL-1 induced T cell activation, and the chemokines MIP-1α, MIP-1β, and MCP-1 recruit immature DC, monocytes, and Th1 cells. Altogether, these data indicate that a subset of PDC gradually upregulates a homodimeric CD8α receptor upon HSV-1 stimulation, exposes a highly activated phenotype, and appears to be particularly active in recruiting other immune cells to the site of inflammation.

## Conclusion

This is—at least to our knowledge—the first report that a subset of human PDC is capable of expressing CD8α at the cell surface upon HSV-1 stimulation. This subset is phenotypically different from the CD8α^–^ PDC in expressing increased levels of markers for activation, costimulation, and migration. In parallel, CD8α^+^ PDC secrete enhanced levels of proinflammatory cytokines and chemokines. Therefore, this subset may play an important role in innate and adaptive immune defenses in HSV-1 infections. So far, it is unclear whether CD8α^+^ PDC are just a more activated subset, which “does better” than CD8α^–^ PDC, or whether they have additional or different functions, such as being actively involved in cross-presentation. Further studies are required to define the conditions under which PDC present antigen efficiently and which formulation of antigen fits best for PDC cross-presentation (Villadangos and Young, 2008). Notably, murine knockouts for IRF8 lead to deficiencies in PDC and lymphoid-resident CD8α^+^ DC (Schiavoni et al., 2002; Aliberti et al., 2003; Tsujimura et al., 2003). This phenomenon may point to a common link in development and possibly function of these two cell populations. Further analyses of human CD8α-expressing PDC will delineate their role in the defense against viral infections, and—if viral vectors are used—also in anti-tumor responses.

## Author Contributions

PS, ST, and MW performed the experiments, JV contributed multiplex cytokine bead array data and performed proof-reading, and BS and PS wrote the manuscript.

### Conflict of Interest Statement

The authors declare that the research was conducted in the absence of any commercial or financial relationships that could be construed as a potential conflict of interest.

## Abbreviations

DC: dendritic cells
HSV: Herpes simplex virus
IFN: interferon
IL: interleukin
PBMC: peripheral blood mononuclear cells
PDC: plasmacytoid dendritic cells.
